# Chromosome Data and Karyotype Diversity of Anurans from Madagascar: Half a Century After the First Broad Cytosystematic Approach

**DOI:** 10.3390/genes16121464

**Published:** 2025-12-08

**Authors:** Marcello Mezzasalma, Gaetano Odierna, Elvira Brunelli, Fabio M. Guarino

**Affiliations:** 1 Department of Biology, Ecology and Earth Science, University of Calabria, Via P. Bucci 4/B, 87036 Rende, Italy; elvira.brunelli@unical.it; 2 Department of Biology, University of Naples Federico II, Via Cinthia 26, 80126 Naples, Italy; gaetanodierna@gmail.com (G.O.); fabguari@unina.it (F.M.G.)

**Keywords:** Anura, chromosome banding, chromosome evolution, cytogenetics, evolution, Madagascar

## Abstract

Madagascar is one of the world’s most prominent biodiversity hotspots and is characterized by exceptionally high amphibian diversity, with 429 currently described, mostly endemic species. However, cytogenetic research on Malagasy amphibians has been conducted only intermittently over the years. Previous studies, mostly using conventional staining and banding methods and often confined to single taxa or isolated families, have provided only partial insights into the karyotype evolution and genome organization of the major Malagasy clades. In this contribution, we present the first comprehensive synthesis of all available cytogenetic data on Malagasy anurans, including chromosome number and morphology, heterochromatin distribution, and chromosomal markers across the major endemic Malagasy families. By integrating and comparing results from decades of scattered studies, this review reveals consistent patterns of chromosomal diversification and identifies evolutionary trends associated with speciation and adaptive radiation in Malagasy amphibians. Overall, native Malagasy amphibian species can be subdivided into two main karyotype groups: the first includes karyotypes with only biarmed chromosomes (*Heterixalus*, *Ptychadena*, *Boophis*, *Mantella*, and *Guibemantis*), while the second comprises karyotypes with one or more uniarmed elements (*Gephyromantis*, *Mantidactylus*, and Microhylidae). The localization of NORs follows a diverse pattern, often varying even among closely related species. Heterochromatin distribution and composition also appear to be species-specific and thus taxonomically informative. Beyond summarizing existing knowledge, this work establishes a unified framework for interpreting chromosome evolution within the unique biogeography and evolutionary history of Madagascar. Our synthesis provides essential baseline data for future molecular, genomic, and conservation studies, thereby enhancing our understanding of the mechanisms that have generated and maintained the island’s extraordinary amphibian diversity.

## 1. Introduction

Madagascar, due to its exceptionally high biodiversity, largely composed of endemic taxa, has long been considered one of the “hottest hotspots” on the planet [1,2,3] with remarkably high levels of endemism for vertebrates, reaching nearly 100% of terrestrial mammal, reptile, and amphibian species [4,5,6].

In particular, the island hosts an extraordinary diversity of amphibian species characterized by distinctive morphological and genomic features, many of which face significant conservation threats (e.g., [3,4,5]).

To date, 429 anuran species have been recorded in Madagascar [6], and only three of them are considered non-endemic: *Hoplobatrachus tigerinus*, *Duttaphrynus melanostictus*, and *Ptychadena mascareniensis*. The first two have been introduced to the island from Asia, while *P. mascareniensis* also occurs on the African mainland [3,7,8].

Blommers-Schlösser and Blanc [9], in their monograph, reported 133 frog species occurring in Madagascar. The current number (429) has more than tripled over the last three decades and is likely still underestimated. In fact, several clades provisionally identified as candidate species await formal taxonomic description [10], and many others remain undiscovered [3,11]. A major contribution to the discovery of new species has come from dedicated field expeditions that began at the end of the last century, combined with the use of molecular, morphological, and bioacoustic approaches (e.g., [4,12,13]).

Overall, the native Malagasy frogs currently belong to four families: Hyperoliidae, Mantellidae, Microhylidae, and Ptychadenidae. The introduced *H. tigerinus* and *D. melanostictus* belong to the families Dicroglossidae and Bufonidae, respectively [6].

Chromosomal studies have proven to be valuable tools for identifying plesiomorphic and apomorphic characters, detecting reproductive barriers, and delineating evolutionary trends among taxa, thereby providing essential insights into genome evolution, speciation processes, and phylogenetic relationships (e.g., [14,15,16,17]). However, cytogenetic research on Malagasy frogs has been conducted intermittently, mainly during the 1970s and the first decade of the 21st century. The first pioneering study was carried out by Morescalchi [18], who described the karyotype of *Mantella aurantiaca*. This was followed by the work of Blommers-Schlösser and Blanc, who described the karyotypes of 54 species representing the four Malagasy families using standard chromosome staining [19,20,21,22]. After a 20-year hiatus, chromosomal research resumed with the study by Pintak et al. [23], who described the karyotype and C-banding pattern of seven *Mantella* species.

Subsequent cytogenetic analyses were carried out in different laboratories and institutions, using conventional staining, banding techniques (Ag-NOR, C-banding, and sequential C-banding with fluorochromes), and NOR-FISH methods. These studies provided chromosomal data for 68 species, 51 of which were analyzed for the first time [15,16,24,25,26,27,28,29,30,31,32].

In this contribution, we provide a comprehensive review of all the available cytogenetic data on Malagasy amphibians by more than doubling the data discussed in the first cytosystematic synthesis by Blommers-Schlösser [22]. We describe and interpret, from an evolutionary perspective, the general karyotype structure, chromosome number and morphology, and the abundance and localization of different chromosomal markers, highlighting major patterns of karyotype evolution and their potential relevance for understanding the evolutionary history of Malagasy amphibians. We define putative plesiomorphic and apomorphic chromosomal states, highlighting consistent patterns of chromosomal diversification in Malagasy amphibians that encompass distinct evolutionary tendencies, including karyotype stasis, the progressive formation of uniarmed elements, and taxon-specific patterns of heterochromatin distribution and NOR localization.

This work is intended as an informative overview of evolutionary cytogenetics on Malagasy amphibians for non-specialists as well as an inclusive summary and reference guide for more experienced researchers.

## 2. Methods

The relevant scientific literature was systematically identified and selected through electronic searches in several databases, including Google Scholar^®^, PubMed^®^Web of Science^®^, and JSTOR^®^ from April 2025 to July 2025. We searched specifically for terms: “amphibians” or “anurans” or “frogs” or “toads” or “Bufonidae” or “Dicroglossidae” or “Hyperoliidae” or “Mantellidae” or “Microhylidae” or “Ptychadenidae” AND “chromosome” or “karyotype” or “cytogenetic” or “karyology” or “cytotaxonomy” AND “Madagascar” or “Malagasy” or “Madagascan”. A supplementary search was performed by reviewing the references cited in the initially retrieved papers and consulting the authors’ personal literature databases.

## 3. Chromosome Number and Morphology

Karyotype data are so far available for 135 native anuran species, representing 31% of the 429 known total Malagasy species (Table 1).

This percentage appears far from the approximately 50% considered by Blommers-Schlösser [22], highlighting that most of the karyological diversity of Malagasy amphibians is still unknown. It should also be considered that in recent years several molecular phylogenetic studies have identified new (often cryptic) species (e.g., [11,33]). Notably, various species have been studied by different authors, and in several instances the karyotypes reported differ, mainly in the number of meta- and submetacentric chromosomes. Besides erroneous taxonomic identifications, these differences could be likely due to different colchicine concentrations and/or exposure time. In fact, the colchicine arrests the cells in metaphase of mitosis while the chromosome condensation proceeds [34]. It is plausible that chromosomes with a centromeric index close to the transition values between different chromosome classes (e.g., meta- and submetacentric) may have been assigned to different classes in different studies.

For completeness, we included in Table 1 the karyotype data of the two anuran species introduced from Asia to Madagascar (*D. melanostictus* and *H. tigerinus*) [8,31,32], but Bufonidae and Dicroglossidae are not naturally present on the island. In particular, the *D. melanostictus* karyotype of 2n = 22 with all biarmed chromosomes, with the first eight pairs relatively larger than the remaining pairs, is the most common karyotype in Bufonidae and it is considered the ancestral chromosome condition in the family [35,36]. Similarly, the karyotype of *H. tigerinus* of 2n = 26, with all biarmed chromosomes and the first five pairs relatively larger than the remaining eight pairs, represents the hypothesized ancestral condition in Ranoidea [37,38].

**Figure 1:**
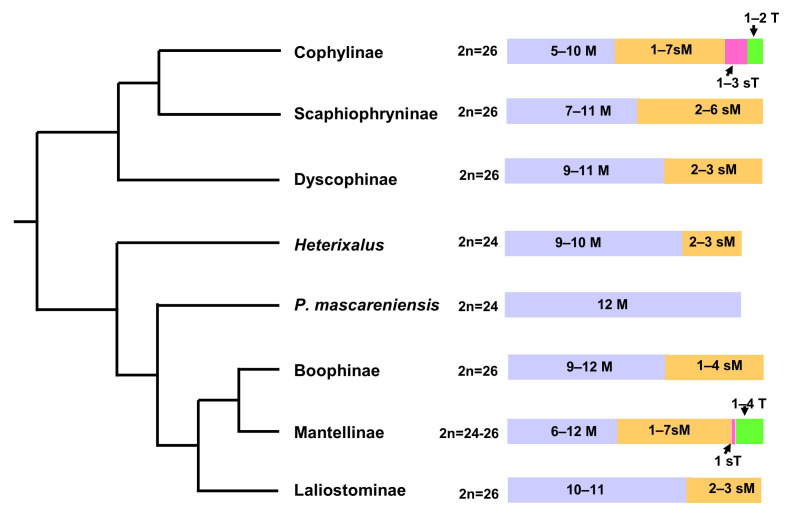
Phylogenetic relationships of major clades of Malagasy anurans retrieved from AmphibiaWeb [6] with described chromosome numbers and variability in number of metacentric (M), submetacentric (sM), subtelocentric (sT), and acrocentric (T) chromosomes.

Considering together all the available chromosome data, it is possible to highlight that karyotype diversity in amphibians from Madagascar is only partially represented by variation in chromosome number (Figure 1).

In particular, concerning chromosome morphology, the native Malagasy amphibian species can be subdivided into two main karyotype groups: the first includes karyotypes with only biarmed chromosomes (*Heterixalus*, *Ptychadena*, *Boophis*, *Mantella*, *and Guibimantis*), while the second comprises karyotypes including one or more uniarmed elements (*Gephyromantis*, *Mantidactylus*, and Microhylidae) (see Table 1) (Figure 1).

The genus *Heterixalus*, as do many other genera of the family Hyperoliidae, shows a remarkable karyological stasis with a karyotype of 2n = 24 with all biarmed chromosomes, which gradually decrease in length [27,38,39]. The origin of the 2n = 24 karyotype of Hyperoliidae is debated. According to Bogart and Tandy [39], it originated once during the split between Raninae (mostly with 2n = 26) and other ranoids. Conversely, according to Morescalchi [38], karyotypes of 2n = 24 have been derived multiple times, independently, from karyotypes of 2n = 26. Morescalchi [38] also suggested that the rearrangements involved pericentromeric inversions originating from acrocentric chromosomes, followed by their fusion and the formation of new centromeres. A third, alternative, and more parsimonious hypothesis involves a tandem fusion between two chromosome pairs of the ancestral 2n = 26, followed by centromere inactivation [40,41].

Similar to *Heterixalus*, the genus *Ptychadena* has a conserved karyotype of 2n = 24 with all biarmed chromosomes [22,39].

Among Mantellidae, a notable karyotype stasis is shown by all Boophinae, Laliostominae, and, among Mantellinae, by all the *Blommersia*, *Guibemantis*, and *Mantella* species with a known karyotype. All these taxa share a karyotype of 2n = 26, all biarmed chromosomes, and with the first five pairs distinctively larger than the other eight pairs (Table 1). Subtle differences have been occasionally described in the number of meta- and submetacentric elements among different clades [16,21,26] (see Table 1), but more focused analyses should be performed to ascertain if they are due to clade-specific chromosome mutations or if they represent research artifacts.

A discrete chromosome variability concerning either chromosome number or morphology can be observed in Microhylidae and, among Mantellidae, in *Gephyiromantis*, *Mantidactylus*, and *Spinomantis*. In fact, among the 32 Microhylidae species so far karyotyped, 14 show a distinct number (1–5) of uniarmed chromosomes (FN = 42, 44, 46, 48 or 50) (see Table 1), which are probably derived from chromosome inversions, centromeric shifts and/or addition/deletion of heterochromatin [42,43,44,45] (see also below).

Inversions represent well-known drivers of chromosome speciation and are able to maintain adaptive divergence between populations, mostly by suppressing recombination in the inverted regions [17,43,46]. In contrast, centromere shift and centromere repositioning do not influence the sequence order in the chromosomes involved, but can severely alter chromosome architecture, influencing the meiotic process with possible implications for speciation events [47].

Interestingly, *Scaphiophryne gottlebei* (Scaphiophryninae) represents the only known polyploid (tetraploid, 2n = 52 = 4x = 13) Malagasy amphibian species so far documented [29]. According to Vences et al. [29], the tetraploid condition of *S*. *gottlebei* likely had an allopolyploid origin, which occurred early during the diversification of the Malagasy microhylids.

Among the four studied species of *Spinomantis*, a reduced number of chromosomes (2n = 24) is shown by *S. aglavei* and *S*. aff. *aglavei*. In *Gephyromantis*, among the twelve species so far karyotyped, seven show a karyotype of 2n = 26 with one or more uniarmed elements (FN ranging from 42 to 50); four have 2n = 26 all biarmed chromosomes, while *G. striatus* presents a karyotype of 2n = 24. In *Mantidactylus*, among the 13 species so far karyologically investigated, five retain the chromosome number of 2n = 26 biarmed elements, three species have 2n = 26 with uniarmed chromosomes (FN = 44, 48, and 50), and the other five species have had the chromosome number reduced to 2n = 24 (Table 1). Overall, these data suggest that three main evolutionary tendencies have characterized the chromosome evolution of Malagasy amphibians: karyotype stasis, reduction of the chromosome number from 2n = 26 to 2n = 24, and the progressive formation of uniarmed chromosomes. Furthermore, in *Mantidactylus*, an increase in the chromosome number has been reported by Aprea et al. [24], probably as the result of a chromosome fission of primitive biarmed elements.

## 4. Nucleolar Organizing Regions (NORs)

The localization of major ribosomal cistrons (NORs) follows a variable pattern in different taxa of Malagasy anurans. Different genera are generally characterized by distinct chromosome localization patterns of NORs, which often also vary among closely related species. In the genera *Boophis*, *Mantella*, and *Heterixalus*, loci of NORs have been invariably identified on the sixth, the second, and the ninth pair, respectively. However, in *Heterixalus*, different species show loci of NORs alternatively on the short arms or the long arms of the ninth pair, suggesting the occurrence of a chromosome inversion or centromeric shift (see Table 1). In *Gephyromantis*, karyotype variability in chromosome morphology (due to the presence of a variable number of uniarmed elements, see above) is also reflected in a variable localization of loci of NORs, which can be alternatively found on the long arm of the 1st, 6th, 8th, 10th or 11th chromosome pair in different species [16,22,30] (see Table 1). In contrast, in Microhylidae, loci of NORs are mostly conserved within genera, but their localization varies among them, namely on the second pair in *Paradoxophyla* and *Scaphiophryne*, on the fourth pair in *Platypelis*, and on the sixth pair in *Anodonthyla*, *Plethodontohyla*, and *Dyscophinae*) (e.g., [21,26]) (see Table 1).

The different chromosomal localization of loci of NORs in both *Gephyromantis* and Microhylidae species suggests that their translocation may result from cryptic translocation events and/or the amplification of distinct ribosomal loci, accompanied by the deletion of relatively ancestral sites in different species [15,16]. Alternatively, the variation in NOR localization within *Gephyromantis* and among different genera of Microhylidae could have arisen from a polymorphic state in their common ancestor, followed by the selective amplification of a specific site in each lineage [48].

The available data on the number and chromosomal localization of NOR loci in other Malagasy amphibian clades are too limited to propose consistent evolutionary scenarios, and further targeted analyses are needed to clarify their distribution patterns and evolutionary dynamics.

## 5. Heterochromatin

The application of fluorochromes in chromosome banding and staining methods for Malagasy amphibians included quinacrine (which detects AT-rich DNA clusters) [36], Chromomycin A_3_ (CMA)/Distamycin DAPI (DA-DAPI), (which reveals the presence of GC- and AT-rich DNA clusters, respectively) [49] and CMA/Methyl Green (CMA/MG) (which evidences GC-rich DNA clusters) [50]. These fluorochromes have been applied individually or in conjunction with C-banding and/or sequential C-banding (e.g., +CMA, +DAPI, and +Giemsa), enabling the investigation of the localization and composition of specific heterochromatin families in native Malagasy anurans and contributing to the elucidation of lineage-specific diversification events (see e.g., [15,16,24,25,26,27,28,29,30]). As generally observed in other amphibian and vertebrate taxa, heterochromatin is preferentially localized on pericentromeric, paracentromeric, and/or telomeric chromosomal regions; however, its chromosomal distribution and composition often result in species-specific differences in several Malagasy amphibian taxa. In fact, heterochromatin patterns greatly differ among species, even in genera showing a general invariability in chromosome number and morphology (with a particular regard to *Boophis*, *Mantella*, and *Heterixalus*), where it has been considered taxonomically relevant (e.g., [21,25,28]).

The role of heterochromatin in speciation is highly debated, but growing evidence indicates that the amplification and deletion of repetitive DNA can have a significant evolutionary impact on the genome structure. In fact, the rapid evolution of repetitive sequences disrupts the interaction between chromosomes and the proteins that bind them, promoting chromosome rearrangements, meiotic mispairing, and contributing to population diversification and speciation [51,52]. As mentioned above, the uniarmed chromosomes observed in several taxa of Malagasy amphibians could have potentially arisen by asymmetrical addition/deletion of large amounts of pericentromeric heterochromatin. Anurans, and amphibians in general, have a plastic genome with large variation in the DNA content (up to 3–4 times even among species of the same genus) [53]. In amphibians, fractions of highly repetitive DNA sequences are usually responsible for large quantitative genome variations [54], but more focused analyses are needed to ascertain if variation in the heterochromatin content can be linked to the appearance of acrocentric elements in *Gephyromantis*, *Mantidactylus*, and Microhylidae.

Attempts in *Boophis*, *Mantella*, and *Heterixalus* failed to consistently test if the heterochromatin variations were phylogenetically significant [25,27,29] because of the highly variable nucleotide composition of this genomic material, which is often constituted by highly repetitive DNA families [55].

The analysis of heterochromatin amount and chromosomal distribution in the karyotype of *S. gottlebei* was instrumental in confirming the allopolyploid origin of tetraploidy in this microhylid species [29]. Tetraploidy can arise through either autopolyploidy or allopolyploidy [17], and the heteromorphic heterochromatic pattern of *S. gottlebei* supports an allopolyploid origin [29]. In fact, polyploidy is generally better tolerated in amphibians than in other tetrapods [17], and can have profound evolutionary consequences, as it creates a meiotic barrier with diploid relatives, effectively enabling instant speciation [56,57]

Interestingly, analysis of chromosome distribution and heterochromatin localization in the genus *Dyscophus* revealed a fixed polymorphism in *D. guineti* on the 11th chromosome pair [15]. This observation led to the hypothesis that this derived chromosomal condition may have acted as a post-zygotic reproductive barrier, whereby hybrids between the rearranged and non-rearranged karyotypes could experience reduced fertility or viability, potentially driving speciation via a sympatric or parapatric route [15].

In the introduced dicroglossid *H. tigerinus*, C-banding staining revealed the presence of a ZW sex chromosome system, with a completely heterochromatic W chromosome [32]. In contrast, similar methods did not reveal the presence of any sex chromosomes in native Malagasy amphibian species, and mechanisms of sex determination should be further investigated to clarify their genetic and evolutionary bases.

## 6. Conclusions

Chromosome diversity and evolution in Malagasy amphibians are still significantly understudied. Karyotype data are, in fact, currently available only for less than one-third of the described species (135 karyotypes and 429 described species). Nevertheless, the available chromosome data have successfully highlighted that several different evolutionary tendencies characterized the karyotype evolution of anurans from Madagascar. Among them, a karyotype evolutionary stasis (mostly concerning chromosome number, morphology, and localization of NOR loci) of a putative ancestral configuration of 2n = 26 chromosomes and all biarmed elements is recognizable in most taxa.

Deviation from this ancestral configuration of chromosome number and morphology mostly involves two different processes. The first is represented by a reduction of the chromosome number (in Mantellinae, Hyperoliinae, and Ptychadenidae), while the second involves the progressive formation of uniarmed elements (1–5) via chromosome inversions, centromeric shifts, or addition/deletion of heterochromatin. We also highlight that heterochromatin distribution patterns and the number and chromosome localization of NORs appear to be taxonomically relevant in different taxa and may have contributed to processes of speciation and lineage diversification.

Future research should prioritize the description of new karyotypes, the characterization of cytogenetic markers with taxonomic significance, and the elucidation of chromosomal evolutionary processes to improve our understanding of their role in speciation and phylogenetic diversification. Furthermore, future studies should incorporate modern genomic methods and molecular cytogenetic approaches, such as high-throughput sequencing, comparative genomic hybridization, chromosome painting, and genome-wide mapping of selected chromosome markers, which will allow a clearer and more comprehensive interpretation of clade-specific chromosome changes.

## Figures and Tables

**Table 1:** Available chromosome data of anurans from Madagascar: M = metacentric, sM = submetacentric, sT = subtelocentric, A = acrocentric, p = chromosome short arm, q = chromosome long arm; AgNOR = AgNOR staining; CB = C-banding; F = fluorochromes; ZW = ZW sex chromosomes.

Family/Subfamily/Genus	Species	Karyotype	Banding/Staining	Reference
**Bufonidae**				[31]
*Duttaphrynus* (30 sp.)	*D. melanostictus*	3n = 22; 8M, 3sM; FN = 44	AgNOR (7p); CB	
				
**Dicroglossidae**				
Dicroglossinae (218 sp.)				
*Hoplobatrachus* (201 sp.)	*H. tigerinus*	2n = 26; 8M, 5sM; FN = 52	Ag-NOR [6q], CB, ZW	[32]
				
**Hyperoliidae**				
Hyperoliinae (196 sp.)				
*Heterixalus* (11 sp.)	*H. alboguttatus*	2n = 24; 9M, 3sM; FN = 48	Ag-NOR [9q], CB, F	[27]
	*H. andrakata*	2n = 24; 9M, 3sM; FN = 48	Ag-NOR [9q], CB, F	[27]
	*H. betsileo*	2n = 24; 9M, 3sM; FN = 48		[21]
	*H. betsileo*	2n = 24; 9M, 3sM; FN = 48	Ag-NOR [9q], CB, F (b)	[27]
	*H. boettgeri*	2n = 24; 9M, 3sM; FN = 48	Ag-NOR [9q], CB, F (b)	[27]
	*H. luteostriatus*	2n = 24; 10M, 2sM; FN = 48	Ag-NOR [9p], CB, F (b)	[27]
	*H. punctatus*	2n = 24; 10M, 2sM; FN = 48	Ag-NOR [9p], CB, F (b)	[27]
	*H. rutenbergi*	2n = 24; 9M, 3sM; FN = 48	Ag-NOR [9p], CB, F (b)	[27]
	*H. variabilis*	2n = 24; 9M, 3sM; FN = 48	Ag-NOR [9q], CB, F (b)	[27]
	*H. tricolor*	2n = 24; 7M, 5sM; FN = 48		
	*H. tricolor*	2n = 24; 9M, 3sM; FN = 48	Ag-NOR [9q], CB, F (b)	[27]
				
**Mantellidae**				
Boophinae (87 sp.)				
*Boophis* (87 sp.)	*B. albilabris*	2n = 26; 12M, 1sM; FN = 52	Ag-NOR [6q], CB, F	[25]
	*B. aff. albilabris*	2n = 26; 10M, 3sM; FN = 52	Ag-NOR [6q], CB, F	[25]
	*B. anjanaharibeensis*	2n = 26; 12M, 1sM; FN = 52	Ag-NOR [6q], CB, F	[25]
	*B. ankaratra*	2n = 26; 12M, 1sM; FN = 52	Ag-NOR [6q], CB, F	[25]
	*B. brachychir*	2n = 26; 12M, 1sM; FN = 52	Ag-NOR [6q], CB, F	[25]
	*B. doulioti*	2n = 26; 10M, 3sM; FN = 52	Ag-NOR [6q], CB, F	[25]
	*B. aff. elenae*	2n = 26; 11M, 2sM; FN = 52	Ag-NOR [6q], CB, F	[25]
	*B. erythrodactylus*	2n = 26; 9M, 4sM; FN = 52		[22]
	*B. erythrodactylus*	2n = 26; 10M, 3sM		[25]
	*B. goudotii*	2n = 26; 11M, 2sM; FN = 52		[22]
	*B. goudotii*	2n = 26; 13M; FN = 52	Ag-NOR [6q], CB, F	[25]
	*B. aff. luteus*	2n = 26; 10M, 3sM; FN = 52	Ag-NOR [6q], CB, F	[25]
	*B. madagascariensis*	2n = 26; 9M, 4sM; FN = 52		[22]
	*B. aff. madagascariensis*	2n = 26; 13M; FN = 52	Ag-NOR [6p], CB, F	[25]
	*B. aff. mandraka*	2n = 26; 12M, 1sM; FN = 52		[22]
	*B. aff. mandraka*	2n = 26; 12M, 1sM; FN = 52	Ag-NOR [6p], CB, F	[25]
	*B. aff. marojezensis*	2n = 26; 12M, 1sM; FN = 52		[22]
	*B. microtympanum*	2n = 26; 13M; FN = 52	Ag-NOR [6q], CB, F	[25]
	*B. occidentalis*	2n = 26; 13M; FN = 52	Ag-NOR [6q], CB, F	[25]
	*B. opisthodon*	2n = 26; 11M, 2sM; FN = 52		[22]
	*B. aff. rappiodes*	2n = 26; 12M, 1sM; FN = 52	Ag-NOR [6q], CB, F	[25]
	*B. reticulatus*	2n = 26; 12M, 1sM; FN = 52	Ag-NOR [6q], CB, F	[25]
	*B. aff. rufioculis*	2n = 26; 12M, 1sM; FN = 52	Ag-NOR [6p], CB, F	[25]
	*B. tephraeomystax*	2n = 26; 8M, 5sM; FN = 52		[22]
	*B. tephraeomystax*	2n = 26; 10M, 3sM; FN = 52		[22]
	*B. aff. septrentrionalis*	2n = 26; 12M, 1sM; FN = 52	Ag-NOR [6q], CB, F	[25]
	*B. viridis*	2n = 26; 12M, 1sM; FN = 52	Ag-NOR [6q], CB, F	[25]
	*B. williamsi*	2n = 26; 9M, 4sM; FN = 52)		[22]
	*B. xerophilis*	2n = 26; 13M; FN = 52)	Ag-NOR [6q], CB, F	[25]
Laliostominanae (7 sp.)				
*Aglyptodactylus* (6 sp.)	*A. madagascariensis*	2n = 26; 11M, 2sM; FN = 52		[22]
*Laliostoma* (1 sp.)	*L. labrosum*	2n = 26, 10M, 3sM; FN =52		[22]
				
Mantellinae (193 sp.)				
*Blommersia* (14 sp.)	*B. blommersae*	2n = 26; 12M, 1sM; FN = 52		[22]
	*B. galani*	2n = 26; 12M, 1sM; FN = 52		[22]
	*B. grandisonae*	2n = 26, 10M, 3sM; FN =52	Ag-NOR [1p], CB, F	[28]
*Boehmantis* (1 sp.)	*B. microtympanum*			
*Gephyromantis* (62 sp.)	*G. asper*	2n = 26; 6M, 3sM, 4A; FN = 44		[22]
	*G. granulatus*	2n = 26; 8M, 4sM, 1A; FN = 50	Ag-NOR [8q], CB, F	[30]
	*G. leucomaculatus*	2n = 26; 6M, 6sM, 1T; FN = 50	Ag-NOR [6q], CB, F	[30]
	*G. luteus*	2n = 26; 6M, 4sM, 1sT, 2A; FN = 46		[22]
	*G. luteus*	2n = 26; 6M, 2sM, 1sT, 4A; FN = 42	Ag-NOR [11q], CB, F	[30]
	*G. aff. moseri*	2n = 26; 8M, 5sM; FN = 52		[30]
	*G. pseudoasper*	2n = 26; 7M, 7sM; FN = 52	Ag-NOR [9q], CB, F	[30]
	*G. redimitus*	2n = 26; 7M, 5sM, 1A; FN = 50	Ag-NOR [6q], CB, F	[30]
	*G. salegy*	2n = 26; 5M, 7sM, 1sT; FN = 50	Ag-NOR [6q], CB, F	[30]
	*G. sp. Ca 19*	2n = 26; 8M, 5sM; FN = 52	Ag-NOR [6q], CB, F	[16]
	*G. striatus*	2n = 24; 6M, 1sM, 5A; FN = 42	Ag-NOR [10q], CB, F	[16]
	*G. zavona*	2n = 26; 9M, 4sM; FN = 52	Ag-NOR [6q], CB, F	[30]
*Guibemantis* (26 sp.)	*G. bicalcaratus*	2n = 26; 11M, 2sM; FN = 52	Ag-NOR [1p], CB, F	[28]
	*G. aff. bicalcaratus*	2n = 26; 9M, 4sM; FN = 52		[22]
	*G. depressiceps*	2n = 26; 10M, 3sM; FN = 52		[22]
	*G. liber*	2n = 26; 10M, 3sM; FN = 52		[22]
	*G. methueni*	2n = 26 11M, 2sM; FN = 52		[22]
	*G. pulcher*	2n = 26; 9M, 4sM; FN = 52		[22]
	*G. punctatus*	2n = 26; 9M, 4sm; FN = 52		[22]
	*G. aff. punctatus*	2n = 26; 10M, 3sM; NF = 52	Ag-NOR [1p], CB, F	[28]
	*G. timidus*	2n = 26; 11M, 2sM; FN = 52		[22]
*Mantella* (16 sp.)	*M. aurantiaca*	2n = 26; 10M, 3sM; FN = 52		[18]
	*M. aurantiaca*	2n = 26; 10M, 3sM; FN = 52		[22]
	*M. aurantiaca*	2n = 26; 11M, 2sM; FN = 52	CB	[23]
	*M. aurantiaca*	2n = 26; 11M, 2sM; FN = 52	Ag-NOR [2p], CB, F	[28]
	*M. baroni*	2n = 26; 10M, 3sM; FN = 52	CB	[23]
	*M. baroni*	2n = 26; 11M, 2sM; FN = 52	Ag-NOR [2p], CB, F	[28]
	*M. betsileo*	2n = 26; 11M, 2sM; FN = 52		[22]
	*M. betsileo*	2n = 26; 11M, 2sM; FN = 52	CB	[23]
	*M. betsileo*	2n = 26; 12M, 1sM; FN = 52	Ag-NOR [2p], CB, F	[28]
	*M. aff*, *betsileo*	2n = 26; 10M, 3sM; FN = 52	Ag-NOR [2p], CB, F	[28]
	*M. cowanii*	2n = 26; 9M, 4sM; FN = 52		[22]
	*M. crocea*	2n = 26; 10M, 3sM; FN = 52	CB	[23]
	*M. expectata*	2n = 26; 10M, 3sM; FN = 52	Ag-NOR [2p], CB, F	[28]
	*M. haraldmeieri*	2n = 26; 8M, 5sM; FN = 52	CB	[23]
	*M. laevigata*	2n = 26; 11M, 2sM; FN = 52	CB	[23]
	*M. laevigata*	2n = 26; 10M, 3sM; FN = 52	Ag-NOR [2p], CB, F	[28]
	*M. madagascariensis*	2n = 26; 11M, 2sM; FN = 52	Ag-NOR [2p], CB, F	[28]
	*M. nigricans*	2n = 26; 10M, 2sM, 1A; FN = 50	Ag-NOR [2p], CB, F	[28]
	*M. pulcra*	2n = 26; 10M, 3sM; FN = 52	Ag-NOR [2p], CB, F	[28]
	*M. viridis*	2n = 26; 11M, 2sM; FN = 52	CB	[23]
	*M. viridis*	2n = 26; 11M, 2sM; FN = 52	Ag-NOR [2p], CB, F	[28]
*Mantidactylus* (58 sp.)	*M. alutus*	2n = 24; 12M; FN = 48	Ag-NOR [6q], CB, F	[28]
	*M. ambohimitombi*	2n = 26; 10M, 3sM; FN = 52		[22]
	*M. aff. auremnalis*	2n = 26; 10M, 2sM, 1A; FN = 50		[22]
	*M. betsileanus*	2n = 24; 6M, 3sM, 1A; FN = 50		[22]
	*M. aff. biporus*	2n = 24; 8M, 4sM; FN = 48		[22]
	*M. cowani*	2n = 26; 12M, 1A; FN = 50	Ag-NOR [6p], CB, F	[16]
	*M. sp.* Ca 19	2n = 24; 7M, 5sM; FN = 48		[22]
	*M. aff. femoralis*	2n = 26; 9M, 3sM, 1A; FN = 50		[22]
	*M. guttulatus*	2n = 26; 10M, 3sM; FN = 52		[22]
	*M. lugubris*	2n = 26; 9M, 3sM, 1A; FN = 52		[22]
	*M. paidroa*	2n = 26; 6M, 7sM; FN = 52		[22]
	*M. sp.* Ca11	2n = 26; 10M, 2sM, 1A; AN = 50		[16]
	*M. aff. ulcerosus*	2n = 24; 8M, 2sM, 1sT, 1A; FN = 44		[22]
*Spinomantis* (14 sp.)	*S. aglavei*	2n = 24; 9M, 3sM; AN = 48		[22]
	*S. aff. aglavei*	2n = 24; 9M, 3sM; FN = 48	Ag-NOR [7q], CB, F	[16]
	*S. peraccae*	2n = 26; 7M, 6M; FN = 52		[22]
	*S. phantasticus*	2n = 26; 13M; NF = 52		[16]
	*S. sp.* Ca3	2n = 26; 12M, 1sM; FN = 52	Ag-NOR [6p], CB, F	[16]
				
**Microhylidae**				
Cophylinae (117 sp.)				
*Anilany* (1 sp.)	*A. helenae*			
*Anodonthyla* (12 sp.)	*A. boulengerii*	2n = 26; 10M, 3sM; FN = 52		[21]
	*A. montana*	2n = 26; 10M, 3sM; FN = 52		[19]
	*A. montana*	2n = 26; 10M, 3sM; FN = 52	Ag-NOR [6p], CB, F	[26]
	*A. moramora*	2n = 26; 10M, 3sM; FN = 52	Ag-NOR [6p], CB, F	[26]
*Cophyla* (7 sp.)	*C. phyllodactyla*	2n = 26; 8M, 4sM; 1A; FN = 50	Ag-NOR [6p], CB, F	[26]
*Madecassophryne* (1 sp.)	*M. truebae*			
*Mini* (3 sp.)				
*Platypelis* (17 sp.)	*P. barbouri*	2n = 26; 9M, 2sM; 2sT; FN = 48		[21]
	*P. grandis*	2n = 26; 9M, 3sM, 1A; FN = 50		[21]
	*P. grandis*	2n = 26; 9M, 2sM, 2A; FN = 48	Ag-NOR {4p], CB, F	[26]
	*P. aff. mavomavo*	2n = 26; 9M, 3sM, 1A; FN = 50	Ag-NOR {4p], CB, F	[26]
	*P. olgae*	2n = 26; 9M, 2sM, 2A; FN = 48		[21]
	*P. pollicaris*	2n = 26; 8M, 4sM, 1sT; FN = 50		[21]
	*P. tuberifera*	2n = 26; 8M, 4sM, 1sT; FN = 50		[21]
	*P. tuberifera*	2n = 26;10M, 2sM, 1A; FN = 50	Ag-NOR {4p], CB, F	[26]
*Plethodontohyla* (12 sp.)	*P. alluaudi*	2n = 26; 7M, 5sM, 1sT; FN = 50		[21]
	*P. alluaudi*	2n = 26; 9M, 2sM, 2sT; FN = 48	Ag-NOR {2q], CB, F	[26]
	*P. brevipes*	2n = 26; 8M, 4sM, 1sT; FN = 50	Ag-NOR {2q], CB, F	[26]
	*P. mihanika*	2n = 26; 10M, 2sM, 1sT; FN = 50	Ag-NOR {6q], CB, F	[26]
	*P. aff. minuta*	2n = 26; 9M, 2sM, 1sT, 1A; FN 48	Ag-NOR {6q], CB, F	[26]
	*P. notosticta*	2n = 26; 5M, 5sM, 3sT; FN = 46		[21]
	*P. tuberata*	2n = 26; 5M, 6sM, 2sT; FN = 48		[21]
	*P. tuberata*	2n = 26; 9M, 2sM, 1sT, 1A; FN 48	Ag-NOR {6q], CB, F	[26]
*Rhombophryne* (20 sp.)	*R. testudo*	2n = 26; 10M, 2sM; 1st; FN = 50	Ag-NOR {2p], CB, F	[26]
*Stumpffia* (44 sp.)	*S. gimmeli*	2n = 26; 10M, 3sM; FN = 52	Ag-NOR {8p], CB, F	[26]
	*S. aff. grandis*	2n = 26; 9M, 4sM; FN = 52	Ag-NOR {6p], CB, F	[26]
	*Stumpffia* sp.	2n = 26; 9M, 3sM; 1A; FN = 50	Ag-NOR {1p], CB, F	[26]
Dyscophinae (3 sp.)				
*Dyscophus* (3 sp.)	*D. antongilii*	2n = 26; 11M, 2sM; FN = 52		[21]
	*D. antongilii*	2n = 26; 9M, 4sM; FN = 52	Ag-NOR [6q], CB, F	[15]
	*D. guineti*	2n = 26; 10M, 3sM; FN = 52		[21]
	*D. guineti*	2n = 26; 9M, 4sM; FN = 52	Ag-NOR [6q], CB, F	[26]
	*D. guineti*	2n = 26; 9M, 4sM; FN = 52		[15]
	*D. insularis*	2n = 26; 10M, 3sM; FN = 52		[21]
	*D. insularis*	2n = 26; 9M, 4sM; FN = 52	Ag-NOR [6q], CB, F	[15]
**Scaphiophryninae**				
*Paradoxophyla* (2 sp.)	*P. palmata*	2n = 26; 10M, 3sM; FN = 52		[21]
	*P. palmata*	2n = 26; 7M, 6sM; FN = 52	Ag-NOR [2p], CB, F	[26]
	*P. tiarano*	2n = 26; 7M, 6sM; FN = 52	Ag-NOR [2p], CB, F	[26]
*Scaphiophryne* (10 sp.)	*S. borybory*	2n = 26; 7M, 6sM; FN = 52	Ag-NOR [2p], CB, F	[26]
	*S. calcarata*	2n = 26; 7M, 6sM; FN = 52	Ag-NOR [2p], CB, F	[26]
	*S. gottlebei*	2n = 52 = 4x = 13; 7M, 6sM; FN = 104	Ag-NOR [2p], CB, F	[26]
	*S. madagascariensis*	2n = 26; 8M, 5sM; FN = 52		[20]
	*S. madagascariensis*	2n = 26; 7M, 6sM; FN = 52	Ag-NOR [2p], CB, F	[29]
	*S. spinosa*	2n = 26; 11M, 2sM; FN = 52	Ag-NOR [2p], CB, F	[29]
				
Ptychadenidae (63 sp.)				
*Ptychadena* (59 sp.)	*P. mascareniensis*	2n = 24; 12M; FN = 48		[22]

## Data Availability

No new data were created or analyzed in this study. Data sharing is not applicable to this article.

## References

[1] Myers N. (1988). Threatened biotas: “Hot spots” in tropical forests. Environmentalist.

[2] Myers N., Mittermeier R.A., Mittermeier C.G., da Fonseca G.A.B., Kent J. (2000). Biodiversity hotspots for conservation priorities. Nature.

[3] Gehring P.-S., Köhler J., Strauß A., Randrianiaina R.D., Glos J., Glaw F., Vences M., Zachos F., Habel J. (2011). The Kingdom of the Frogs: Anuran Radiations in Madagascar. Biodiversity Hotspots.

[4] Andreone F., Vences M., Randrianirina J.E. (2001). Patterns of amphibian and reptile diversity at Berara Forest (Sahamalaza Peninsula), NW Madagascar. Ital. J. Zool..

[5] IUCN 2025 The IUCN Red List of Threatened Species. Version 2025-2.

[6] AmphibiaWeb. 2025. University of California, Berkeley, CA, USA. https://amphibiaweb.org.

[7] Vences M., Brown J.L., Lathrop A., Rosa G.M., Cameron A., Crottini A., Dolch R., Edmonds D., Freeman K.L.M., Glaw F. (2017). Tracing a toad invasion: Lack of mitochondrial DNA variation, haplotype origins, and potential distribution of introduced *Duttaphrynus melanostictus* in Madagascar. Amphibia-Reptilia.

[8] Guarino F.M., Andreone F., Mezzasalma M., Licata F., Puoti S., Santos B., Cocca W., Solofoniaina Fidy J.F., Ndriantsoa S.H., Noel J. (2023). Life History Traits and Longevity of the Invasive Asian Common Toad *Duttaphrynus melanostictus* (Schneider, 1799) in Madagascar. Animals.

[9] Blommers-Schlösser R.M.A., Blanc C.P. (1991). Amphibiens (premie’re partie). Faune Madag..

[10] Vences M., Wake D.B., Heatwole H.H., Tyler M. (2007). Speciation, species boundaries and phylogeography of amphibians. Amphibian Biology.

[11] Cocca W., Andreone F., Belluardo F., Rosa G.M., Randrianirina J.E., Glaw F., Crottini A. (2020). Resolving a taxonomic and nomenclatural puzzle in mantellid frogs: Synonymization of *Gephyromantis azzurrae* with *G. corvus*, and description of *Gephyromantis kintana* sp. nov. from the Isalo Massif, western Madagascar. ZooKeys.

[12] Vences M., Glaw F., Böhme W. (1999). A review of the genus Mantella (Anura, Ranidae, Mantellinae): Taxonomy, distribution and conservation of Malagasy poison frogs. Alytes.

[13] Glaw F., Vences M., Lourenço W.R., Goodman S.M. (2000). Current counts of species diversity and endemism of Malagasy amphibians and reptiles. Diversité et Endémisme a Madagascar.

[14] Rieseberg L.H. (2001). Chromosomal rearrangements and speciation. Trends Ecol. Evol..

[15] Mezzasalma M., Andreone F., Aprea G., Glaw F., Odierna G., Guarino F.M. (2017). When can chromosomes drive speciation? The peculiar case of the Malagasy tomato frogs (genus *Dyscophus*). Zool. Anz..

[16] Mezzasalma M., Andreone F., Odierna G., Guarino F.M.G., Crottini A. (2022). Comparative cytogenetics on eight Malagasy Mantellinae (Anura, Mantellidae) and a synthesis of the karyological data on the subfamily. Comp. Cytogenet..

[17] Mezzasalma M., Brunelli E., Odierna G., Guarino F.M. (2023). Evolutionary and Genomic Diversity of True Polyploidy in Tetrapods. Animals.

[18] Morescalchi A. (1967). Le relazioni tra il cariotipo di anuri diplasioceli. Caryologia.

[19] Blommers R. (1971). Karyotype de Anodonthyla montana Angel (Anura, Microhylidae) du massif de l’Andringitra (Madagascar). Terre Malg..

[20] Blommers R., Blanc C.P. (1972). Existence d’une variability chromosomique intrasp6cifique chez *Pseudohemisus madagascariensis* Blgr. (Batraciens, Ranid6s). Bull. Soc. Zool. Ft..

[21] Blommers-Schlösser R.M.A. (1976). Chromosomal analysis of twelve species of Microhylidae (Anura) from Madagascar. Genetica.

[22] Blommers-Schlösser R.M.A. (1978). Cytotaxonomy of the Ranidae, Rhacophoridae, Hyperoliidae (Anura) from Madagascar with a note on the Karyotype of two amphibians of the Seychelles. Genetica.

[23] Pintak T., Vences M., Glaw F., Böhme W. (1998). Comparative chromosome morphology of Malagasy poison frogs (Amphibia: Ranidae: *Mantella*). Folia Zool..

[24] Aprea G., Andreone F., Capriglione T., Odierna G. Chromosome banding in several Malagasy anuran species belonging to the genera *Aglyptodactylus*, *Boophis* and *Mantidactylus*. Proceedings of the 9th Ordinary General Meeting of the Societas Herpetologica Europaea.

[25] Aprea G., Andreone F., Capriglione T., Odierna G., Vences M. (2004). Evidence for a remarkable stasis of chromosome evolution in Malagasy treefrogs (*Boophis*, Mantellidae). Ital. J. Zool..

[26] Aprea G., Odierna G., Andreone F., Glaw F., Vences M. (2007). Karyological evolution and systematics of Malagasy microhylid frogs. Zool. Anz..

[27] Odierna G., Aprea G., Andreone F., Böhme W., Vences M. (2007). Cytosystematics of hyperoliid frogs: Phylogeny of *Heterixalus*, low karyotypic variability in hyperoliines and separate phylogenetic position of Leptopelis. Ital. J. Zool..

[28] Odierna G., Vences M., Aprea G., Lötters S., Andreone F. (2001). Chromosome data for Malagasy poison frogs (Amphibia: Ranidae: *Mantella*) and their bearing on taxonomy and phylogeny. Zool. Sci..

[29] Vences M., Aprea G., Capriglione T., Andreone F., Odierna G. (2002). Ancient tetraploidy and slow molecular evolution in *Scaphiophryne*: Ecological correlates of speciation mode in Malagasy relict amphibians. Chrom. Res..

[30] Andreone F., Aprea G., Vences M., Odierna G. (2003). A new frog of the genus *Mantidactylus* from the rainforests of north-eastern Madagascar, and its karyological affinities. Amphibia-Reptilia.

[31] Saba N., Tripathi N.K., Bolwan W.K. (2014). Karyotypic study of the common Indian toad, *Duttaphrynus melanostictus*, from Jammu And Kashmir, India. RW-IRSSJ.

[32] Saba N. (2014). Tripathi. Preliminary cytogenetic study and report of ZZ/ZW sex chromosomes in the bullfrog, *Hoplobatrachus tigerinus* (Anura, Amphibia) from high altitude area of Jammu and Kashmir, India. Nucleus.

[33] Perl R.B., Nagy Z.T., Sonet G., Glaw F., Wollenberg K.C., Vences M. (2014). DNA barcoding Madagascar’s amphibian fauna. Amphibia-Reptilia.

[34] Levan A. (1939). The effect of colchicine on meiosis in Allium. Hereditas.

[35] Bogart J.P., Blair W.F. (1972). Karyotypes. Evolution of Genus Bufo.

[36] Schmid M. (1978). Chromosome banding in Amphibia, I. Constitutive heterochromatin and nucleolus organizer regions in *Bufo* and *Hyla*. Chromosoma.

[37] Schmid M. (1978). Chromosome banding in Amphibia, II. Constitutive heterochromatin and nucleolus organizer regions in Ranidae, Microhylidae and Rhacophoridae. Chromosoma.

[38] Morescalchi A. (1981). Karyology of the main groups of African frogs. Monit. Zool. Ital. Suppl..

[39] Bogart J.P., Tandy M. (1981). Chromosome lineages in African ranoid frogs. Monit. Zool. Ital..

[40] Marshall O.J., Chueh A.C., Wong L.H., Choo K.H.A. (2008). Neocentromeres: New insights into centromere structure, disease development, and karyotype evolution. Am. J. Hum. Genet..

[41] Han Y.H., Zhang Z.H., Liu C.X., Liu J.H., Huang S.W.M., Jin W.W. (2007). Centromere repositioning in cucurbit species. Implication of the genome impact from centromere activation and inactivation. Proc. Natl. Acad. Sci. USA.

[42] King M., Chordata, John B., Gwent C. (1990). Amphibia. Animal Cytogenetics.

[43] Coyne J.A., Meyers W., Crittenden A.P., Sniegowski P. (1993). The fertility effects of pericentric inversions in *Drosophila melanogaster*. Genetics.

[44] Ayala D., Ullastres A., González J. (2014). Adaptation through chromosomal inversions in *Anopheles*. Front. Genet..

[45] Allshire R.C., Madhani H.D. (2018). Ten principles of heterochromatin formation and function. Nat. Rev. Mol. Cell Biol..

[46] Faria R., Johannesson K., Butlin R.K., Westram A.M. (2018). Evolving inversions. Trends Ecol. Evol..

[47] Guo X., Su H., Shi Q., Fu S., Wang J., Zhang X., Hu Z., Han F. (2016). De Novo Centromere Formation and Centromeric Sequence Expansion in Wheat and its Wide Hybrids. PLoS Genet..

[48] Gama J.M., Gazolla C.B., de Souza D.Y., Recco-Pimentel S.M., Bruschi D.P. (2019). Recurrent variation in the active NOR sites in the monkey frogs of the genus *Pithecopus* Cope, 1866 (Phyllomedusidae, Anura). Comp. Cytogenet..

[49] Schweizer D. (1981). Counterstain enhanced chromosome banding. Hum. Genet..

[50] Sahar E., Latt S.A. (1980). Energy transfer and binding competition between dyes used to enhance staining differentiation in metaphase chromosomes. Chromosoma.

[51] Thakur J., Packiaraj J., Henikoff S. (2021). Sequence, Chromatin and Evolution of Satellite DNA. Int. J. Mol. Sci..

[52] Liao X., Zhu W., Zhou J., Li H., Xu X., Zhang B., Gao X. (2023). Repetitive DNA sequence detection and its role in the human genome. Commun. Biol..

[53] Schmid M., Steinlein C., Feichtinger W., Poot M. (1993). Chromosome banding in Amphibia: XVIII. Karyotype evolution and genomic size variation in *Pleurodema* (Anura, Leptodactylidae). Cytogenet. Cell Genet..

[54] Olmo E. (1991). Genome variations in the transition from amphibians to reptiles. J. Mol. Evol..

[55] Verma R.S. (1988). Heterochromatin: Molecular and Structural Aspects.

[56] Mable B.K., Alexandrou M.A., Taylor M.I. (2011). Genome duplication in amphibians and fish: An extended synthesis: Polyploidy in amphibians and fish. J. Zool..

[57] Madlung A. (2013). Polyploidy and its effect on evolutionary success: Old questions revisited with new tools. Heredity.

